# Endoscopic Recognition of Colonic Cystic Lymphangiomatosis: A Rare Benign Finding

**DOI:** 10.14309/crj.0000000000002167

**Published:** 2026-06-11

**Authors:** Sanjeevani Tomar, Nooria Saif, Dennis Yang, Maham Hayat

**Affiliations:** 1 Department of Gastroenterology and Hepatology, AdventHealth Orlando, Orlando, FL; 2 Allama Iqbal Medical College, Lahore, Pakistan; 3 Center for Interventional Endoscopy, AdventHealth Orlando, Orlando, FL

## CASE REPORT

**Figure 1.**
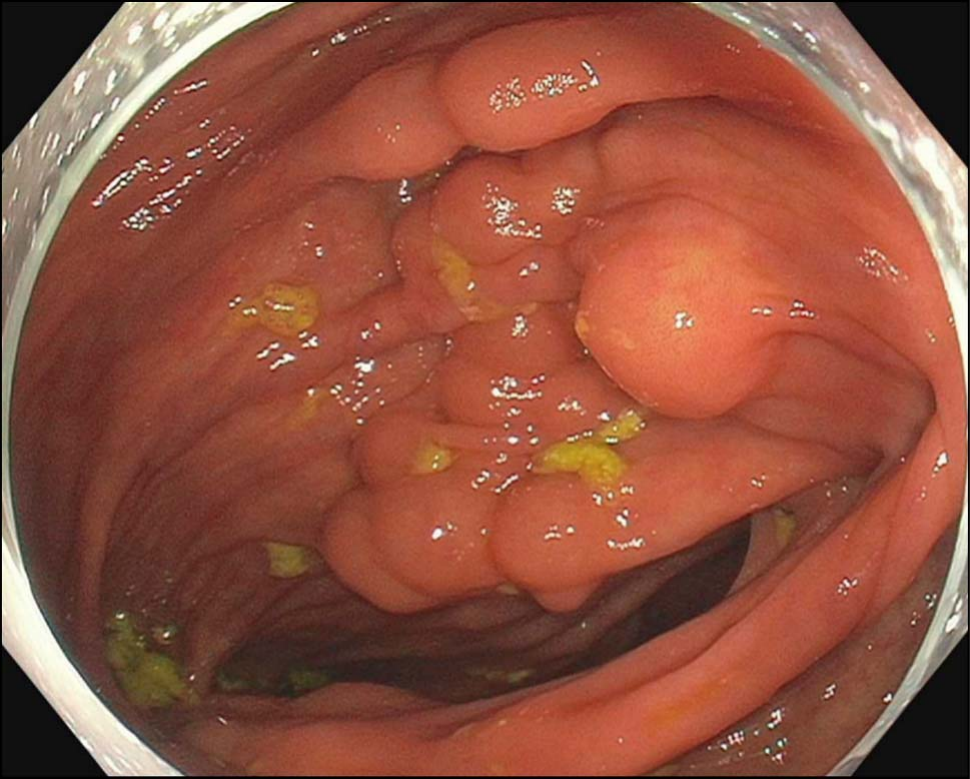
White light endoscopic image showing multiple translucent, cystic submucosal nodules in the ascending colon.

**Figure 2.**
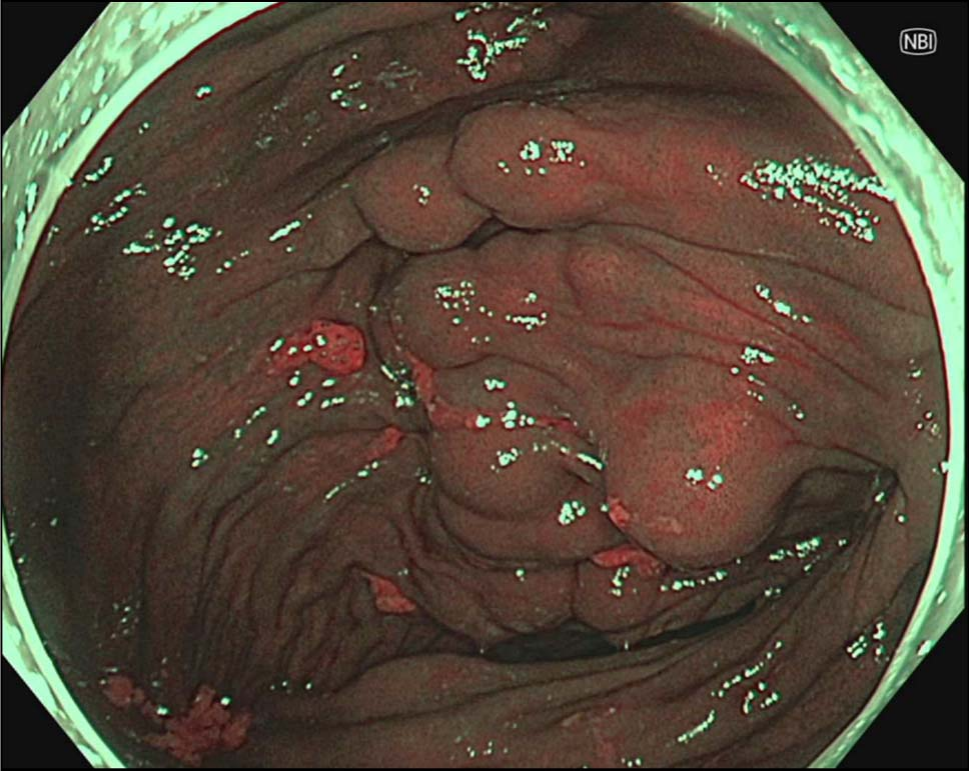
Narrow band imaging showing preserved mucosal and vascular architecture overlying the submucosal nodules.

A 69-year-old man with hypertension and hyperlipidemia underwent a screening colonoscopy revealing multiple submucosal nodules in the ascending colon. The patient was asymptomatic, and prior biopsies showed benign colonic mucosa. Repeat evaluation demonstrated multiple 1–2-cm soft, compressible submucosal nodules with a translucent, cystic appearance (Figure 1). The overlying mucosa was intact without ulceration, erythema, or adenomatous changes. Narrow band imaging revealed preserved mucosal and vascular patterns (Figure 2), without dilated or serpiginous vessels to suggest a vascular etiology such as colonic varices.

**Figure 3.**
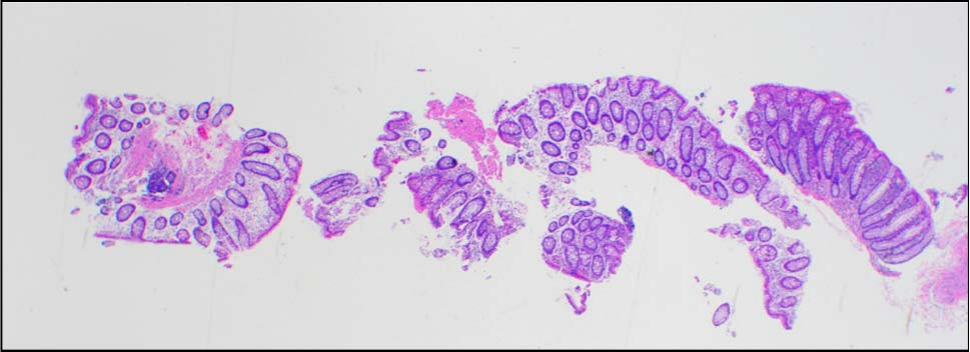
Hematoxylin and eosin stain at 2× magnification showing multiple fragments of colonic mucosa without evidence of dysplasia or malignancy.

Given these features, the lesions were considered low suspicion for vascular origin, and cold forceps biopsies were obtained to exclude mucosal pathology, including dysplasia or malignancy. Histopathology showed benign colonic mucosa without significant abnormalities (Figure 3).

The endoscopic impression was consistent with colonic cystic lymphangiomatosis, a rare benign lymphatic malformation. This condition is typically discovered incidentally and is often asymptomatic, although it can occasionally present symptoms such as bleeding or obstruction.^1–4^ Lymphatic involvement is submucosal, making superficial biopsies often nondiagnostic; definitive diagnosis may require histopathologic confirmation, endoscopic ultrasound, or resection, typically in symptomatic cases.^5^

In this case, diagnosis was based on characteristic endoscopic findings with benign histology, and no intervention was pursued given the absence of symptoms. Although EUS (mini-probe) may support the diagnosis, it is primarily diagnostic and may not be necessary when features are classic.

## DISCLOSURES

Author contributions: S. Tomar: Literature review, drafting and revising the manuscript. N. Saif: Revised for intellectual content. D. Yang: Edit and revised the article for intellectual content. M. Hayat: Provided the images, critically reviewed the article, and is the article guarantor.

Financial disclosure: Dennis Yang, MD, FACG: Consultant for Boston Scientific, Fujifilm, Olympus, Medtronic, Microtech, 3D-Matrix, and Neptune Medical. Received research support from 3D-Matrix and Microtech.

Informed consent was obtained for this case report.
